# Genetic and Environmental Risk Factors Associated With Trajectories of Depression Symptoms From Adolescence to Young Adulthood

**DOI:** 10.1001/jamanetworkopen.2019.6587

**Published:** 2019-06-28

**Authors:** Alex S. F. Kwong, José A. López-López, Gemma Hammerton, David Manley, Nicholas J. Timpson, George Leckie, Rebecca M. Pearson

**Affiliations:** 1 Medical Research Center Integrative Epidemiology Unit, University of Bristol, Bristol, United Kingdom; 2 School of Geographical Sciences, University of Bristol, Bristol, United Kingdom; 3 Centre for Multilevel Modelling, University of Bristol, Bristol, United Kingdom; 4 Population Health Sciences, Bristol Medical School, University of Bristol, Bristol, United Kingdom; 5 Centre for Academic Mental Health, University of Bristol, Bristol, United Kingdom; 6 School of Education, University of Bristol, Bristol, United Kingdom

## Abstract

**Importance:**

Less favorable trajectories of depressive mood from adolescence to early adulthood are associated with current and later psychopathology, impaired educational attainment, and social dysfunction, yet the genetic and environmental risk factors associated with these trajectories are not fully established. Examining what risk factors are associated with different trajectories of depressive mood could help identify the nature of depression symptoms and improve preventive interventions for those at most risk.

**Objective:**

To examine the differential associations of genetic and environmental risk factors with trajectories of depression symptoms among individuals observed from ages 10 to 24 years.

**Design, Setting, and Participants:**

In a longitudinal cohort study established in 1990 and currently ongoing (the Avon Longitudinal Study of Parents and Children [ALSPAC]), growth mixture modeling was used to identify trajectories of depression symptoms in 9394 individuals in the United Kingdom. Associations of different risk factors with these trajectories were then examined. Analysis was conducted between August 2018 and January 2019.

**Main Outcomes and Measures:**

Trajectories were composed from depression symptoms measured using the Short Mood and Feelings Questionnaire at 9 occasions from ages 10 to 24 years. Risk factors included sex, a polygenic risk score taken from a recent genome-wide association study of depression symptoms, maternal postnatal depression, partner cruelty to the offspring’s mother when the child was aged 2 to 4 years, childhood anxiety at age 8 years, and being bullied at age 10 years.

**Results:**

Data on all risk factors, confounders, and the outcome were available for 3525 individuals, including 1771 (50.2%) who were female. Trajectories were assessed between the mean (SD) age of 10.7 (0.3) years and mean (SD) age of 23.8 (0.5) years. Overall, 5 distinct trajectories of depression symptoms were identified: (1) stable low (2506 individuals [71.1%]), (2) adolescent limited (325 individuals [9.2%]), (3) childhood limited (203 individuals [5.8%]), (4) early-adult onset (393 individuals [11.1%]), and (5) childhood persistent (98 individuals [2.8%]). Of all the associations of risk factors with trajectories, sex (odds ratio [OR], 6.45; 95% CI, 2.89-14.38), the polygenic risk score for depression symptoms (OR, 1.47; 95% CI, 1.10-1.96), and childhood anxiety (OR, 1.30; 95% CI, 1.16-1.45) showed the strongest association with the childhood-persistent trajectory of depression symptoms compared with the stable-low trajectory. Maternal postnatal depression (OR, 2.39; 95% CI, 1.41-4.07) had the strongest association with the early-adult–onset trajectory, while partner cruelty to mother (OR, 2.30; 95% CI, 1.36-3.90) had the strongest association with the adolescent-limited trajectory. Bullying (OR, 8.08; 95% CI, 4.92-13.26) showed the strongest association with the childhood-limited trajectory.

**Conclusions and Relevance:**

The least favorable trajectories of depression symptoms (childhood persistent and early-adult onset) were associated with both genetic and environmental risk factors, but the 2 trajectories of limited duration that had resolved by early adulthood (childhood limited and adolescent limited) were not associated with the polygenic risk score or maternal postnatal depression. Bullying was strongly associated with both the childhood-persistent and childhood-limited trajectories, suggesting that this risk factor may have a time-specific effect. These findings suggest that examining genetic and multiple time-specific environmental antecedents could help identify trajectories of varying onset and chronicity.

## Key Points

**Question:**

Are genetic and environmental risk factors associated with different trajectories of depression symptoms during adolescence and young adulthood?

**Findings:**

In a cohort study of 3525 individuals observed from ages 10 to 24 years, both genetic and environmental risk factors were associated with childhood-persistent and early-adult–onset trajectories of depression symptoms, while adolescent-limited and childhood-limited trajectories were not associated with genetic risk factors.

**Meaning:**

Differential patterns of timing and the nature of genetic and environmental risk factors were associated with different trajectory groups for depression symptoms, which could help to guide the timing and focus of prevention strategies.

## Teaser

This cohort study examines the differential associations of genetic and environmental risk factors with trajectories of depression symptoms among individuals observed from ages 10 to 24 years in the Avon Longitudinal Study of Parents and Children (ALSPAC).

## Introduction

Depression is a leading cause of disability worldwide^1^ and is expected to be the highest global burden of disease by 2030.^2^ Despite efforts to improve interventions, prevalence is still increasing, especially in adolescence.^3^ Evidence suggests that depression during adolescence is associated with many concurrent and later psychological and social impairments.^4,5^ However, what is driving this increase in adolescent depression is still not clear. A greater understanding of the nature of adolescent depression and how to minimize it is crucial if we are to reduce this global burden.

There is evidence that depression should be viewed on a continuum^6,7^ because individuals with subthreshold depression^8,9^ and elevated levels of depression symptoms^10^ are also at risk of concurrent and later psychopathology. Importantly, a similar pattern is also observed for those displaying consistently higher levels of depression symptoms over time.^11,12,13,14,15^ Detailed longitudinal analysis provides an opportunity to further understand not only how depression may manifest differentially over time but also its etiology. Toward this goal, researchers have identified trajectories of depression symptoms during adolescence and potential risk factors associated with those trajectories.^16,17^ Previous research suggests that adolescence is characterized by a clear increase in symptoms of depressed mood; however, trajectories vary within the population and differ by age at onset as well as duration and severity of symptoms.^12,16,17,18^ Evidence has shown that several less favorable trajectories of depression symptoms (ie, those with depression symptoms that start high and continue [childhood persistent], those that start low but increase over time [early-adult onset], or those that start high in early childhood but decline during adolescence and young adulthood [childhood limited]) are present across multiple populations and often associated with poorer outcomes compared with adolescents with low symptoms over time (ie, the stable-low trajectory).^11,12,19,20^ It may be possible to start targeting specific interventions and treatments for certain individuals by disentangling the risk factors (or combinations of risk factors) of different trajectories.

Identifying how different risk factors are associated with varying patterns of depressive mood could be important for understanding the etiology of depression and improving treatment. Risk factors such as sex,^14,19^ childhood psychopathology,^11,20^ parental mood,^19,21^ and early-life socioeconomic position^11,22^ are important factors of less favorable trajectories of depression symptoms. Stronger associations are typically observed for chronically high or increasing trajectories,^11,18,19,20,21^ yet the evidence is not clear cut. A study by Rice et al^23^ also found that polygenic risk for depression was associated with a late-adolescence–onset trajectory, implying that genetic liability may be a key component for less favorable trajectories at specific periods of development.^24^ The evidence is less clear on what might affect trajectories limited to adolescence or childhood, although research suggests they could be reactive to more immediate stressors and events.^11^ For example, bullying is arguably among the strongest factors in adolescent and adult depression^25,26,27^ and is most frequent and salient during certain periods of childhood and adolescent development. Therefore, bullying in childhood is likely to have immediate consequences. However, the direction of this association is unknown, and there is evidence that bullying is also associated with depression throughout the life course, suggesting it could also be reflective of preexisting susceptibility.^28^ Therefore, it is unclear whether the association of bullying with depression is time specific, dependent on other prior factors, or both.^29^ Investigating how bullying affects differential trajectories of depressive symptoms across adolescent development could give insight into how and when the effect of this risk factor occurs. Bullying in childhood has also yet to be linked to different trajectories of depressive symptoms.

Trajectories of depression symptoms are likely to have a highly complex etiology composed of genetic and environmental influences. This is because behavioral phenotypes, such as depression, are unlikely to have purely direct genetic or environmental pathways.^30^ Instead, it is more likely that an interplay of genetic-environmental correlations exists and the contribution of genetic or environmental factors may be greater for complex traits (such as depression).^7,27,31,32,33^ For example, stressful life events may cause more severe depression symptoms, but it is possible that genetically liable individuals may be more prone to stressful life events, thus making it hard to determine the direction of effects. Therefore, while we cannot yet separate whether a risk factor operates through genetic or environmental mechanisms, examining both genetic and environmental risk factors could build better prediction models and provide a new understanding that could be translated into improved prevention and interventions.

To our knowledge, no studies have examined the longitudinal nature of trajectories of depression symptoms in adolescence or their associations with genetic and environmental risk factors in early childhood and adolescence. Understanding whether different risk profiles are associated with specific manifestations of trajectories of depression symptoms may offer more precise opportunities to target interventions during certain periods. We hypothesized that the most high-risk trajectories (ie, childhood persistent) would be associated with a combination of genetic and environmental risk factors (possibly reflecting a complex genetic-environmental interplay and/or a greater genetic contribution, in which genetic liability is reinforced by environmental events). However, given the ambiguity surrounding the antecedents of adolescent-limited and childhood-limited trajectories, there may be some specificity in these trajectories that could be more reflective of emotional reactions to recent negative environmental events, such as bullying.

## Methods

### Study Sample

We used data from the Avon Longitudinal Study of Parents and Children (ALSPAC), a longitudinal cohort study that recruited pregnant women residing in Avon, United Kingdom, with expected delivery dates of April 1, 1991, to December 31, 1992.^34,35^ The initial cohort consisted of 14 062 children. Ethical approval was obtained from the ALSPAC Ethics and Law Committee and the local research ethics committees. Participants provided written informed consent to the collection and use of these data. Data in this study were fully anonymized. The study website contains details of the data that are available through a fully searchable data dictionary and variable search tool.^36^ This report follows the Strengthening the Reporting of Observational Studies in Epidemiology (STROBE) reporting guideline.^37^

### Depression Symptoms

Self-reported depression symptoms were measured on 9 occasions using the Short Mood and Feelings Questionnaire (SMFQ)^38^ when participants were aged 10 to 24 years. The SMFQ is a 13-item questionnaire that measures the presence of depression symptoms in the last 2 weeks. It was administered via mail or in clinics. Each item is scored from 0 to 2, resulting in a summed score from 0 to 26. The SMFQ correlates highly with clinical depression^39,40^ and has been used to explore trajectories of depression symptoms in other studies.^41,42^ Descriptive information can be found in eTable 1 in the Supplement.

### Risk Factors

Sex (male vs female) was identified from birth notifications around the time of delivery. The polygenic risk score (PRS) for depression symptoms was created using summary statistics from a recent genome-wide association study (GWAS) on depression symptoms.^43^ The PRS was created by weighting the effect sizes of 120 422 single-nucleotide polymorphisms associated with depression symptoms from the initial GWAS at a *P* < .50 threshold. The PRS was standardized to have a mean of 0 and a standard deviation of 1; thus, a higher PRS represents higher liability to depression symptoms. Maternal postnatal depression (yes vs no) was assessed at 8 weeks post partum, when the mother completed the Edinburgh Postnatal Depression Scale.^44^ A cutoff score of 13 or higher, which indicates probable depression, was used.^45,46^ Partner cruelty to the mother (yes vs no) was assessed by the Family Adversity Index, which asked the mother whether her partner was abusive toward her when the child was aged 2 to 4 years.^22^ Childhood anxiety was measured at approximately age 8 years by asking the child’s main caregiver about the child’s general anxieties, using questions taken from the Development and Well-being Assessment.^47^ A weighted summary score of 0 to 12 was created from 6 questions on childhood anxiety, with 12 indicating maximum anxiety. Childhood bullying was measured using the modified Bullying and Friendship Interview Schedule when the child visited a research clinic at approximately age 10 years.^48^ We used a binary variable (yes vs no) to assess bullying of the child in the last 6 months. Further information on these variables can be found in the eAppendix in the Supplement.

### Confounders

The following confounders were included based on previous literature that associated them with the risk factors and the depression trajectories^41,42,45^: maternal age at birth (in years), maternal socioeconomic status at birth (manual vs nonmanual occupation), maternal educational attainment at birth, parity (first born vs second born vs third born or more), and the first 5 principle components of ancestry to control for population stratification in the genetic analysis. These confounders were not under investigation as hypothesized primary risk factors but were included to examine the effect of confounders on the association of the risk factors under investigation with depression symptoms as well as to mark missing data and participant demographic characteristics.

### Statistical Analysis

We conducted growth mixture modeling (GMM) in Mplus version 8 (Muthén and Muthén)^49^ to identify latent trajectories of depression symptoms using 9 measures of the SMFQ. Briefly, GMM stratifies individuals from a population into multiple heterogeneous trajectories (or latent classes).^50^ Odds ratios (ORs) and their corresponding 95% CIs were derived from multinomial logistic regressions, where the trajectory with the largest sample size was used as the reference. Multinomial logistic regressions were used to calculate *P *values. Statistical significance was set at *P* < .05, and all tests were 2-tailed. Further details about model fit and how we assessed the validity of these trajectories can be found in the eAppendix in the Supplement.

Missing data in the GMM were handled using full-information maximum-likelihood estimation.^49^ We included individuals in the analysis if they had depression symptoms measured on at least 1 occassion.^23^ Previous research on this data has demonstrated little difference on the shape of trajectories, distribution of trajectory membership, or associations of trajectories with outcomes when comparing individuals with at least 1 measurement of depression symptoms with participants with at least 3 measurements or all 9 measurements.^15^

## Results

Data were available for 9394 individuals with at least 1 measurement of depression symptoms. Demographic characteristics for these individuals can be found in eTable 2 in the Supplement, but briefly, individuals included in the study were more likely to be female and have mothers with more education and higher socioeconomic status at birth. The sample size of individuals with all risk factors and confounders was 3525, including 1771 female participants (50.2%). The mean (SD) age at the first measure of depression symptoms was 10.7 (0.3) years, and the mean (SD) age at the last measure of depression symptoms was 23.8 (0.5) years. Results from our GMM indicated that a 5-class trajectory solution was best suited the data (eTable 3 in the Supplement). The shapes of these trajectories and class distributions did not differ substantially between the sample of 9394 individuals and the sample of 3525 individuals (eTables 4-6 in the Supplement).

### Trajectories of Depression Symptoms

**Figure 1.**
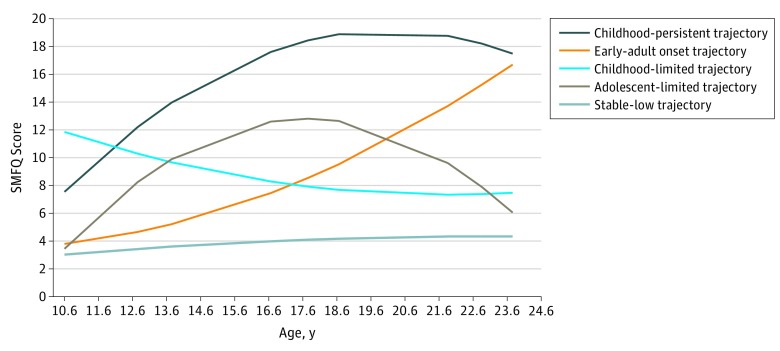
Trajectories of Depression Symptoms From a 5-Class Solution Among the sample of 3525 individuals, 2506 individuals (71.1%) belonged to the stable-low trajectory, 393 (11.1%) belonged to the early-adult–onset trajectory, 325 (9.2%) belonged to the adolescent-limited trajectory, 203 (5.8%) belonged to the childhood-limited trajectory, and 98 (2.8%) belonged to the childhood-persistent trajectory. SMFQ indicates Short Mood and Feelings Questionnaire.

Among the sample of 3525 individuals, 5 heterogeneous trajectories of depression symptoms were derived. First, the stable-low trajectory (2506 individuals [71.1%]) included individuals who had consistently low levels of depression symptoms. Second, the early-adult–onset trajectory (393 individuals [11.1%]) included individuals who started with low depression symptoms that increased during adolescence and young adulthood. Third, the adolescent-limited trajectory (325 individuals [9.2%]) included individuals who experienced elevated levels of depression symptoms only during adolescence, and fourth, the childhood-limited trajectory (203 individuals [5.8%]) included individuals who started with elevated levels of depression symptoms in childhood that decreased. Fifth, the childhood-persistent trajectory (98 individuals [2.8%]) included individuals with moderate levels of depression symptoms that continued to increase and stay high during adolescence and into young adulthood (Figure 1).

### Association of Risk Factors With Trajectories of Depression Symptoms

**Figure 2.**
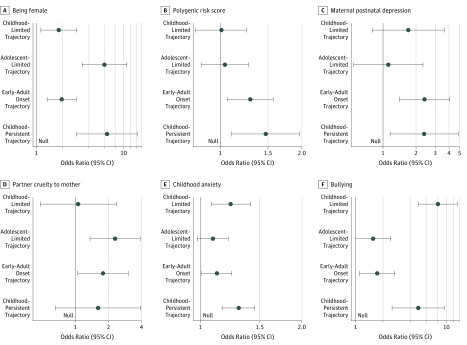
Association of Risk Factors With Trajectories of Depression Symptoms A, Sex was coded 0 for men and 1 for women. Being female was associated with higher risk of all trajectories. B, The polygenic risk score was standardized to have a mean of 0 and an SD of 1. A higher PRS for depression symptoms was associated with the childhood-persistent and early-adult–onset trajectories but not the adolescent-limited or childhood-limited trajectories. C, Maternal postnatal depression was coded 0 for no and 1 for yes. It was associated with the childhood-persistent and early-adult–onset trajectories but not the adolescent-limited or childhood-limited trajectories. D, Partner cruelty to mother was coded as 0 for no and 1 for yes. It was associated with the early-adult–onset and adolescent-limited trajectories but not with the childhood-persistent or the childhood-limited trajectories. E, Childhood anxiety was measured using questions taken from the Development and Well-being Assessment with a summary score ranging from 0 to 12, with higher scores indicating greater childhood anxiety. It was associated with the childhood-persistent, early-adult–onset, and childhood-limited trajectories but not with the adolescent-limited trajectory. F, Bullying was coded as 0 for no and 1 for yes. Being bullied was associated with all 4 at-risk trajectories.

Full results from the multivariate analysis of risk factors with varying trajectories are presented in the Table. The risk factors of varying trajectories of depression symptoms are shown in Figure 2. For the following analysis, the OR represents each trajectory compared with the stable-low trajectory. Correlations between risk factors are shown in eTable 7 in the Supplement, and results did not differ in univariate or unadjusted multivariate analyses (eTable 8 and eTable 9 in the Supplement).

**Table.** Fully Adjusted Multivariate Associations of Risk Factors With Trajectories of Depression Symptoms Among 3525 Individuals

Risk Factor	Multinomial OR (95% CI)^a^				*P* Value
	Childhood Persistent vs Stable Low	Early-Adult Onset vs Stable Low	Adolescent Limited vs Stable Low	Childhood Limited vs Stable Low	
Being female	6.45 (2.89-14.38)	1.96 (1.33-2.88)	6.04 (3.35-10.87)	1.81 (1.13-2.90)	<.001
Polygenic risk score	1.47 (1.10-1.96)	1.29 (1.06-1.57)	1.04 (0.85-1.27)	1.01 (0.81-1.25)	.01
Early life					
Maternal postnatal depression	2.37 (1.16-4.85)	2.39 (1.41-4.07)	1.12 (0.54-2.31)	1.70 (0.80-3.62)	.005
Partner cruelty to mother from ages 2-4 y	1.61 (0.66-3.95)	1.78 (1.05-3.04)	2.30 (1.36-3.90)	1.06 (0.48-2.37)	.008
Childhood					
Anxiety at 8 y	1.30 (1.16-1.45)	1.12 (1.01-1.24)	1.09 (0.98-1.21)	1.23 (1.08-1.41)	<.001
Bullied at 10 y	4.91 (2.52-9.58)	1.73 (1.10-2.70)	1.56 (1.00-2.44)	8.08 (4.92-13.26)	<.001

Abbreviation: OR, odds ratio.

^a^ Analysis was adjusted for all risk factors and the following confounders: maternal age at birth, maternal socioeconomic status at birth, maternal educational attainment at birth, and parity.

Being female was associated with all trajectories (childhood persistent: OR, 6.45; 95% CI, 2.89-14.38; early-adult onset: OR, 1.96; 95% CI, 1.33-2.88; adolescent limited: OR, 6.04; 95% CI, 3.35-10.87; and childhood limited: OR, 1.81; 95% CI, 1.13-2.90). A higher PRS for depression symptoms was associated with the childhood-persistent trajectory (OR, 1.47; 95% CI, 1.10-1.96) and the early-adult–onset trajectory (OR, 1.29; 95% CI, 1.06-1.57) but not the adolescent-limited trajectory (OR, 1.04; 95% CI, 0.85-1.27) or childhood-limited trajectory (OR, 1.01; 95% CI, 0.81-1.25). Maternal postnatal depression was associated with the childhood-persistent trajectory (OR, 2.37; 95% CI, 1.16-4.85) and the early-adult–onset trajectory (OR, 2.39; 95% CI, 1.41-4.07) but not the adolescent-limited trajectory (OR, 1.12; 95% CI, 0.54-2.31) or childhood-limited trajectory (OR, 1.70; 95% CI, 0.80-3.62). Partner cruelty to the mother was associated with the early-adult–onset trajectory (OR, 1.78; 95% CI, 1.05-3.04) and adolescent-limited trajectory (OR, 2.30; 95% CI, 1.36-3.90), but it was not associated with the childhood-persistent trajectory (OR, 1.61; 95% CI, 0.66-3.95) or the childhood-limited trajectory (OR, 1.06; 95% CI, 0.48-2.37). Childhood anxiety was associated with the childhood-persistent trajectory (OR, 1.30; 95% CI, 1.16-1.45), early-adult–onset trajectory (OR, 1.12; 95% CI, 1.01-1.24), and childhood-limited trajectory (OR, 1.23; 95% CI, 1.08-1.41), but it was not associated with the adolescent-limited trajectory (OR, 1.09; 95% CI, 0.98-1.21). Being bullied was also associated with all 4 at-risk trajectories (childhood persistent: OR, 4.91; 95% CI, 2.52-9.58; early-adult onset: OR, 1.73; 95% CI, 1.10-2.70; adolescent limited: OR, 1.56; 95% CI, 1.00-2.44; and childhood limited: OR, 8.08; 95% CI, 4.92-13.26).

## Discussion

This study identified 5 distinct trajectories of depression symptoms from late childhood to young adulthood. These trajectories were associated with both genetic and environmental risk factors. These findings suggest that examining both genetic and environmental antecedents could help identify groups with severe and chronic depression symptoms (ie, in the childhood-persistent trajectory) who should be prioritized for early intervention. Certain risk profiles also showed specific associations with longitudinal patterns of depression symptoms.

Polygenic risk for depression symptoms (ie, greater genetic liability to depression) was associated with the childhood-persistent trajectory and early-adult–onset trajectory, which supports the notion that genetic liability may play an important role in the onset of depression in adolescence.^23,51^ Polygenic risk has previously been associated with both depression and higher trajectories of depression symptoms in later adult populations.^52,53^ However, our results suggest that genetic liability to greater depression symptoms may begin to manifest in childhood and adolescence, as previous research has highlighted.^23^ As such, genetic liability could be a mechanism for chronic and/or more severe depression symptoms throughout the life course that operates through specific neurological or hormonal systems at certain stages of development.^24^ Similar results have been observed for trajectories of attention-deficit/hyperactivity disorder,^54^ suggesting that genetic liability can affect the development of a trait. However, it is unlikely that genetic liability alone is responsible for the more severe trajectories. Instead, a more plausible explanation is that a complex interplay between genetics and the environment exists.^30,31,51^ This is not yet understood, and it is also not clear how genetic liability to depression might affect later environmental risk factors.^55^ Future research should explore this to discover potential pathways and mechanisms involved in the maintenance of depression.

Previous research has shown that bullying is an important factor in the onset of depression in adolescence and adulthood.^25,26^ However, our findings highlight that being bullied in childhood is associated with both short-term and long-term consequences. Whether exposure to bullying has a lasting effect may depend on genetic liabilities to depression and bullying.^27,28,29^ Bullying was assessed at age 10 years (shortly before the first assessment of depression symptoms) and was most associated with the childhood-limited trajectory, which showed severe depression symptoms in early childhood that diminished over time. This could reflect an immediate reaction to bullying that then resolves. Thus, bullying may be a time-specific factor because it had weaker associations with the early-adult–onset trajectory and adolescent-limited trajectory. However, it was associated with the childhood-persistent trajectory, suggesting there could be long-term consequences for individuals who were bullied in childhood but still have consistently severe depression symptoms more than 10 years later. The difference between these 2 trajectories could be that the individuals with the childhood-persistent trajectory also have genetic and additional environmental factors that make it harder to recover. This supports previous research highlighting genetic liability to schizophrenia, and subsequent bullying is associated with worse trajectories of mental health.^56^ This suggests that individuals who experience bullying who have familial and other accumulating risk factors should be prioritized for intervention. A similar interpretation could exist for childhood anxiety, which showed associations with the childhood-persistent trajectory, early-adult–onset trajectory, and childhood-limited trajectory. Interventions to build resilience early for those with genetic liability may yield the most effective method for the prevention of long-term or severe depression.

Sex differences have consistently been associated with less favorable trajectories of depression symptoms,^57^ and we observed strong associations of being female with the childhood-persistent trajectory and the adolescent-limited trajectory, supporting previous research.^19^ The childhood-persistent trajectory and adolescent-limited trajectory are distinct, yet we may have observed these differences because young women who belong to the childhood-persistent group have a genetic liability to depression in addition to other factors, such as early pubertal timing^57,58^ and stress reactivity.^59^ In contrast, the young women with the adolescent-limited trajectory may not have the genetic liability to depression symptoms and are perhaps only reacting to early pubertal timing or stressful events. However, it is likely that other biological and environmental risk factors underlie less favorable trajectories for girls and young women, and more research is needed to disentangle this association.

Maternal postnatal depression was associated with the childhood-persistent trajectory and early-adult–onset trajectory, likely reflecting the transmission of maternal depression to offspring.^60,61,62,63^ This association likely reflects genetic influences (as these 2 trajectories were associated with the PRS) as well as parental depression possibly affecting brain development in utero.^62,64^ Alternatively, it could reflect childhood susceptibilities established in infancy and early development that result in later depression.^63^ Because maternal postnatal depression was not associated with the adolescent-limited trajectory or childhood-limited trajectory (ie, trajectories not associated with genetic liability), it may suggest that maternal depression has more long-lasting associations, possibly through a genetic and environmental interplay. Interestingly, this pattern was not observed for partner cruelty to the mother, which was associated with the early-adult onset and adolescent-limited trajectories but not the childhood-persistent or childhood-limited trajectories (ie, the trajectories associated with bullying). It could be that partner cruelty operates through a different pathway and does not share the same time-specific effect compared with bullying. Instead, the adolescent-limited trajectory could be reflective of depression symptoms that decrease after adolescence, once young adults are less influenced by family life. However, partner cruelty to the mother is a rare exposure, and it is possible that the associations of partner cruelty to the mother with the early-adult–onset and adolescent-limited trajectories reflect a lack of statistical power rather than a true association.

### Limitations

This study has limitations. Previous research has validated the 5-class trajectory model by observing that different trajectories correspond with a diagnosis of depression that is applicable to each time (ie, childhood limited had a stronger association with an early diagnosis, and early-adult onset had a stronger association with a later diagnosis of depression).^15^ Other longitudinal cohorts using similar methods^65,66^ also observed 5-class trajectories, thus increasing the generalizability of these trajectories. However, it is worth noting that previous research using the same data (albeit only to age 18 years)^23^ only identified 3 trajectories of depression symptoms compared with the 5 in this study. This is likely a result of the additional 3 measurement occasions and of the continuous scale of depression symptoms we used compared with the binary diagnosis used by Rice et al.^23^ Despite these methodological differences, our results were broadly the same; genetic liability to depression was associated with a later-onset trajectory and weakly associated with an early-onset trajectory. Additionally, we used genetic and environmental risk factors with more longitudinal data on depression symptoms to explore the long-term associations of these risk factors. However, a problem with longitudinal cohort studies relates to attrition, and although our model used full-information maximum-likelihood estimation to account for missing data, the data could be missing not at random, potentially leading to biased results. Furthermore, the interplay between genetics and the environment is highly complex because genetic and environmental risk factors are not independent (as highlighted by previous research^31^ and correlations between genetic and environmental risk factors in eTable 7 in the Supplement). It is therefore hard to disentangle genetic liability from later environmental risk factors that could be on the causal pathway.

Our analysis using genetic data was also restricted to nonrelated individuals and individuals of European descent. Previous research has shown racial/ethnic differences in trajectories of depression symptoms, with black, Asian, and Hispanic populations all showing less favorable trajectories.^67,68^ We were unable to explore trajectories in these populations owing to the availability of DNA samples from the depressive symptoms GWAS and exclusion criteria, thus our results lack generalizability to these populations.^43^ However, GWASs are being applied to other non-European populations, so future work will be able to untangle racial/ethnic differences in depression research using genetic and environmental determinants.^69^

It is also important to note that we made multiple comparisons, and confidence intervals in many cases overlapped. Therefore, the specificity of the associations deserves replication. Future research that also untangles genetic-environmental correlations will be pivotal for identifying pathways and mechanisms underlying various forms of depression. This will eventually lead to the improvement and treatment of depression.

## Conclusions

In this cohort study, we identified 5 trajectories of depression symptoms from late childhood and young adulthood. The childhood-persistent and early-adult onset trajectories were associated with genetic and environmental risk factors. These findings suggest that looking at the combination of genetic and environmental antecedents could help identify groups with chronic and severe depression symptoms that should be prioritized for intervention. Yet the severity and course of these symptoms could depend on the timing of specific risk factors and other prior susceptibilities. For example, bullying was strongly associated with the childhood-persistent and childhood-limited trajectories, suggesting it may be time specific and that the severity and duration of depression could depend on other factors (ie, genetic liability). Overall, these findings imply that the etiology for trajectories of depression symptoms is multifactorial, with a complex interplay of genetic and environmental contributions. It may be possible to differentiate between risk profiles that show varying trajectories of depression symptoms, which could be transformed into the development of interventions and treatments for those at the most risk of more severe depression symptoms, which could in turn prevent or reduce depression and other detriments in later life.

## Notes

Supplement. **eAppendix. **Supplemental Methods **eTable 1. **Descriptive Statistics and Reliability of the Short Mood and Feelings Questionnaire (SMFQ) **eTable 2.** Participant Demographic Characteristics for Individuals Included in the Analysis **eTable 3.** Growth Mixture Modelling Results Using Different Class Solutions **eTable 4.** Showing Class Counts for the Latent Classes Based on Estimated Posterior Probabilities for Each Model **eTable 5.** Participant Demographic Characteristics for the 5-Class Trajectories Model With No Risk Factors **eTable 6.** Participant Demographic Characteristics for the 5-Class Trajectories Model With All Risk Factors Included **eTable 7.** Matrix of Correlations Between Risk Factors **eTable 8.** Adjusted Univariate Associations of All Risk Factors With Trajectories of Depressive Symptoms **eTable 9.** Unadjusted Multivariate Associations of All Risk Factors With Trajectories of Depressive Symptoms **eReferences**
