# Supplementary material for: Genetic and Environmental Risk Factors Associated With Trajectories of Depression Symptoms From Adolescence to Young Adulthood
Source: JAMA Netw Open. 2019 Jun 28;2(6):e196587. doi: 10.1001/jamanetworkopen.2019.6587 (PMC6604106; doi:10.1001/jamanetworkopen.2019.6587)
Supplement: Supplement. — eAppendix. Supplemental Methods eTable 1. Descriptive Statistics and Reliability of the Short Mood and Feelings Questionnaire (SMFQ) eTable 2. Participant Demographic Characteristics for Individuals Included in the Analysis eTable 3. Growth Mixture Modelling Results Using Different Class Solutions eTable 4. Showing Class Counts for the Latent Classes Based on Estimated Posterior Probabilities for Each Model eTable 5. Participant Demographic Characteristics for the 5-Class Trajectories Model With No Risk Factors eTable 6. Participant Demographic Characteristics for the 5-Class Trajectories Model With All Risk Factors Included eTable 7. Matrix of Correlations Between Risk Factors eTable 8. Adjusted Univariate Associations of All Risk Factors With Trajectories of Depressive Symptoms eTable 9. Unadjusted Multivariate Associations of All Risk Factors With Trajectories of Depressive Symptoms eReferences [file jamanetwopen-2-e196587-s001.pdf]

Supplementary Online Content

Kwong ASF, López-López JA, Hammerton G, et al. Genetic and environmental risk factors associated with trajectories of depression symptoms from adolescence to young adulthood. *JAMA Netw Open*. 2019;2(6):e196587. doi:10.1001/jamanetworkopen.2019.6587

eAppendix. Supplemental Methods

eTable 1. Descriptive Statistics and Reliability of the Short Mood and Feelings Questionnaire (SMFQ)

eTable 2. Participant Demographic Characteristics for Individuals Included in the Analysis

eTable 3. Growth Mixture Modelling Results Using Different Class Solutions

eTable 4. Showing Class Counts for the Latent Classes Based on Estimated Posterior Probabilities for Each Model

eTable 5. Participant Demographic Characteristics for the 5-Class Trajectories Model With No Risk Factors

eTable 6. Participant Demographic Characteristics for the 5-Class Trajectories Model With All Risk Factors Included

eTable 7. Matrix of Correlations Between Risk Factors

eTable 8. Adjusted Univariate Associations of All Risk Factors With Trajectories of Depressive Symptoms

eTable 9. Unadjusted Multivariate Associations of All Risk Factors With Trajectories of Depressive Symptoms

eReferences

This supplementary material has been provided by the authors to give readers additional information about their work.

© 2019 Kwong ASF et al. *JAMA Network Open.*

eAppendix. Supplemental Methods

Biological sex

Biological sex was identified from birth notifications taken around the time of delivery and was coded as male or female. Data for 4 people is missing thus reducing our maximum analytical sample to 9394 (even though individuals with at least one measurement of depressive symptoms were 9398).

Polygenic risk score for depressive symptoms (PRS)

Participants were genotyped using the Illumina HumanHap550 quad chip. Individuals were excluded based on gender mismatches, minimal or excessive heterozygosity, disproportionate levels of individual missingness (>3%), evidence of cryptic relatedness (>10% of alleles identical by descent), insufficient sample replication (IBD < 0.8) and being of non-European ancestry (assessed by multidimensional scaling analysis including HapMap 2 individuals). Thus, our analysis is only on individuals of European descent. SNPs with a minor allele frequency (MAF) of < 1%, Impute2 information quality metric of < 0.8, a call rate of < 95% or evidence for violations of Hardy-Weinberg equilibrium (P-value < 5e-7) were removed. Imputation performed using Impute v2.2.2 with the 1000 genomes reference panel (Phase 1, Version 3), using 2186 reference haplotypes. The maximum number of single nucleotide polymorphisms (snps) that were imputed (and passed filtering on MAF of > 1% and info score > 80%) was 8282911. In the case of siblings, one individual was dropped from analysis in order not to inflate the genetic effect, thus all results are based upon singletons.

The PRS for depressive symptoms was created in PRSice, [1] using summary statistics from a recent genome wide association study (GWAS) of depressive symptoms on 161,460 individuals. [2] We included snps that had a MAF of > 1% and info score > 80%) and excluded SNPs with an R^2 of >0.1, which were within 250Kb of each other. We excluded snps located in the extended MHC region (chromosome 6 (26-33Mb)). Polygenic risk scores were created at various p-value thresholds (between $5x10^{-8}$ and 0.5) and we used the most liberal threshold (0.5) for prediction based upon recent evidence that more liberal polygenic scores may be better predictors if the scores are only concerned with maximising prediction. [3,4]

Population stratification can be a problem in analysis utilising polygenic risk scores, thus, to account for this we adjusted our analysis for the first five principal components of ancestry, as per previous studies. [3]

Childhood bullying

Childhood bullying was measured using the modified Bullying and Friendship Interview Schedule. [5] A child was classed as an overt victim, if he/she was on the receiving end of any of the following five components of overt bullying frequently (several times a month) or very frequently (several times a week):

1. Had personal belongings taken
2. Been threatened/blackmailed
3. Been hit/beaten up
4. Been tricked in a nasty way
5. Been called bad/nasty names

Children who responded with seldom or never to having been bullied for each of the four questions were categorised as not being victims. In addition, children for whom no more than two questions were missing with the remaining items being seldom/never were classed as NOT being bullied.

Growth Mixture Modelling

Trajectories of depressive symptoms were estimated with intercept, slope and quadratic growth factors for each class. Previous work has suggested that quadratic growth may be useful when modelling the non-linearity of trajectories of depressive symptoms. [6-8] To reduce convergence issues, the growth factor variances were constrained to be equal across trajectories. [9] We built a stepwise model starting with a single trajectory (*k*-class) and continued to add trajectories into the model (*k*+1) until the optimal number of trajectories was reached. To assess this optimum, we used a range of criteria including: lowest sample-size-adjusted Bayesian Information Criterion (ssaBIC; which based on the log-likelihood penalised for model complexity as captured by the number of parameters [10]), and hypothesis testing to help model choice by comparing model fit for *k* versus *k*-1 trajectories, using the adjusted likelihood ratio test (LRT) proposed by Lo-Mendell-Rubin. [11] After determining the optimal number of trajectories, covariates (risk factors) were added into the model to examine risk factors for varying trajectory membership. We then compared whether the inclusion of these risk factors affected the overall shape and sample distribution of these trajectories. During this analysis, a bias-adjusted 3-step approach was used, which takes into account the uncertainty in the classification of participants into each trajectory. [12]

© 2019 Kwong ASF et al. *JAMA Network Open.*

eTable 1. Descriptive Statistics and Reliability of the Short Mood and Feelings Questionnaire (SMFQ)

Occasion	Mean Age	Sample Size	Mean SMFQ	SMFQ SD	Above SMFQ Threshold (≥11)	α	Source of SMFQ
1	10.65	7,364	4.04	3.51	5.96%	0.797	Clinic
2	12.81	6,716	3.97	3.86	7.10%	0.842	Clinic
3	13.84	6,019	4.92	4.49	11.66%	0.865	Clinic
4	16.68	4,997	5.91	5.64	18.05%	0.908	Questionnaire
5	17.84	4,497	6.59	5.25	21.64%	0.897	Clinic
6	18.65	3,335	6.83	5.93	21.86%	0.906	Questionnaire
7	21.95	3,305	5.70	5.58	18.06%	0.915	Questionnaire
8	22.88	3,856	6.21	5.55	18.80%	0.906	Questionnaire
9	23.80	3,915	7.03	6.06	24.75%	0.913	Questionnaire

α: Coefficient alpha estimate of reliability for the SMFQ at each occasion. The SMFQ ranges between 0-26 and scores of, or exceeding 11 have been proposed as good markers for depression (see Turner et al., 2014).

© 2019 Kwong ASF et al. *JAMA Network Open.*

**eTable 2.** Participant Demographic Characteristics for Individuals Included in the Analysis (had at least one measurement of depression)			
	Included in Analysis	Excluded in Analysis	x^2, p
**Sex (n=14,854)**			
Males n (%)	4,495 (47.9)	3,140 (57.5)	x^2 = 128.98, p = < .001
Females n (%)	4,899 (52.1)	2,320 (42.5)	
**Maternal Education (n=12,493)**			
A Level or Higher n (%)	3,453 (40.9)	957 (23.7)	x^2 = 566.51, p = < .001
O Level n (%)	2,380 (35.3)	1,347 (33.3)	
< O Level n (%)	2,016 (23.8)	1,740 (43.0)	
**Maternal Socioeconomic Status (n=10,118)**			
Professional/Managerial/Technical n (%)	2,940 (40.8)	841 (28.8)	x^2 = 126.95, p = < .001
Skilled non-manual or lower n (%)	4,263 (59.2)	2,074 (71.2)	
**Parity (n=13,124)**			
First Born n (%)	3,918 (45.9)	1,955 (42.5)	x^2 = 54.16, p = < .001
Second Born n (%)	3,041 (35.7)	1,547 (33.7)	
Third Born + n (%)	1,569 (18.4)	1,094 (23.8)	
**Maternal Age At Pregnancy (n=14,076)**			
< 25 Years n (%)	1,531 (17.3)	1,830 (35.2)	x^2 = 660.82, p = <.001
25-29 n (%)	2,752 (31.0)	1,587 (30.5)	
30-34 n (%)	3,201 (36.1)	1,272 (24.4)	
35+ n (%)	1,388 (15.6)	515 (9.9)	

Pearson's chi-squared tests (x^2) used to highlight differences between participant demographics and inclusion into the study (measured by having at least one measure of depressive symptoms to derive trajectories).
Numbers vary due to missing data on demographics.
No risk factors were included in this analysis.

© 2019 Kwong ASF et al. *JAMA Network Open.*

eTable 3. Growth Mixture Modelling Results Using Different Class Solutions

k	NP	ssaBIC	Entropy	LRT(*p*)
1	10	253634.62	-	-
2	11	252306.66	0.793	<.0001
3	15	251346.88	0.743	<.0001
4	19	250909.03	0.752	.1040
**5**	**23**	**250484.48**	**0.736**	**.0014**
6	27	250230.41	0.735	.0966

k: number of classes; NP: number of parameters; ssaBIC: sample size adjusted Bayesian Information Criterion; LRT: Lo-Mendell-Rubin likelihood ratio test; BLRT.

© 2019 Kwong ASF et al. *JAMA Network Open.*

eTable 4. Showing Class Counts for the Latent Classes Based on Estimated Posterior Probabilities for Each Model

	Childhood Persistent	Early-Adult Onset	Adolescent Limited	Childhood Limited	Stable low
5-Class Model Without Risk Factors or Confounders (n=9,394)	324 (3.5)	1086 (11.6)	880 (9.4)	480 (5.1)	6624 (70.5)
Unadjusted Univariate 5-Class Model (n=4,092)	106 (2.6)	461 (11.3)	393 (9.6)	241 (5.9)	2891 (70.7)
Adjusted Univariate 5-Class Model (n=3,525)	98 (2.8)	393 (11.1)	325 (9.2)	203 (5.8)	2506 (71.1)

% are given in parenthesis.

© 2019 Kwong ASF et al. *JAMA Network Open.*

eTable 5. Participant Demographic Characteristics for the 5-Class Trajectories Model With No Risk Factors

	Childhood Persistent	Adolescent Limited	Early-Adult Onset	Childhood Limited	Stable Low	x^2, p
**Sex (n=9,394)**						
Males n (%)	54 (19.8)	232 (34.6)	203 (27.5)	177 (45.5)	3,829 (52.3)	x^2 = 314.62, p = < .001
Females n (%)	219 (80.2)	439 (65.4)	535 (72.5)	212 (54.5)	3,494 (47.7)	
**Maternal Education (n=8,449)**						
A Level or Higher n (%)	78 (32.0)	259 (42.3)	261 (39.1)	140 (40.4)	2,715 (41.3)	x^2 = 14.09, p = .079
O Level n (%)	89 (36.5)	217 (35.4)	252 (37.7)	124 (35.7)	2,298 (34.9)	
< O Level n (%)	77 (31.5)	137 (22.3)	155 (23.2)	83 (23.9)	1,564 (23.8)	
**Maternal Socioeconomic Status (n=7,203)**						
Professional/Managerial/Technical n (%)	73 (36.5)	201 (39.3)	227 (40.7)	103 (35.3)	2,336 (40.4)	x^2 = 6.53, p = .163
Skilled non-manual or lower n (%)	127 (63.5)	310 (60.7)	331 (59.3)	189 (64.7)	3,306 (58.6)	
**Parity (n=8,528)**						
First Born n (%)	95 (38.8)	288 (47.4)	299 (44.7)	166 (47.2)	3,070 (46.2)	x^2 = 12.53, p = .129
Second Born n (%)	91 (37.1)	220 (36.2)	229 (34.2)	124 (35.2)	2,377 (35.7)	
Third Born + n (%)	59 (24.1)	100 (16.4)	141 (21.1)	62 (17.6)	1,207 (18.4)	
**Maternal Age At Pregnancy (n=8,872)**						
< 25 Years n (%)	54 (21.0)	126 (19.7)	116 (16.7)	79 (21.7)	1,156 (16.7)	x^2 = 18.31, p = .107
25-29 n (%)	67 (26.1)	199 (31.2)	207 (29.9)	94 (25.8)	2,185 (31.6)	
30-34 n (%)	97 (37.7)	218 (34.2)	251 (36.2)	134 (36.8)	2,501 (36.1)	
35+ n (%)	39 (15.1)	95 (14.9)	119 (17.2)	57 (15.7)	1,078 (15.6)	

Pearson's chi-squared tests (x^2) used to highlight differences between participant demographics and the varying trajectories of depression symptoms.
No risk factors were included in this analysis.

© 2019 Kwong ASF et al. *JAMA Network Open.*

**eTable 6.** Participant Demographic Characteristics for the 5-Class Trajectories Model With All Risk Factors Included						
	Childhood Persistent	Adolescent Limited	Early-Adult Onset	Childhood Limited	Stable Low	x^2, p
**Sex (n=4,092)**						
Males n (%)	23 (23.7)	113 (39.8)	87 (26.8)	84 (44.9)	1,729 (54)	x^2 = 130.74, p = < .001
Females n (%)	74 (76.3)	171 (60.2)	237 (73.2)	103 (55.1)	1,471 (46)	
**Maternal Education (n=4,041)**						
A Level or Higher n (%)	37 (38.5)	144 (50.9)	154 (48)	88 (48.1)	1,477 (46.8)	x^2 = 8.62, p = .375
O Level n (%)	35 (36.5)	94 (33.2)	119 (37.1)	60 (32.8)	1,108 (35.1)	
< O Level n (%)	24 (25.0)	45 (15.9)	48 (14.9)	35 (19.1)	573 (18.1)	
**Maternal Socioeconomic Status (n=3,567)**						
Professional/Managerial/Technical n (%)	36 (40.9)	113 (45.9)	135 (48.9)	69 (43.7)	1,265 (45.2)	x^2 = 2.35, p = .671
Skilled non-manual or lower n (%)	52 (59.1)	133 (54.1)	141 (51.1)	89 (56.3)	1,534 (54.8)	
**Parity (n=4,034)**						
First Born n (%)	38 (39.2)	129 (46.2)	139 (43.6)	87 (47.0)	1,476 (46.8)	x^2 = 4.95, p = .763
Second Born n (%)	41 (42.3)	104 (37.3)	115 (36)	65 (35.2)	1,140 (36.1)	
Third Born + n (%)	18 (18.6)	46 (14.5)	65 (20.4)	33 (17.8)	538 (17.1)	
**Maternal Age At Pregnancy (n=4,092)**						
< 25 Years n (%)	15 (15.5)	33 (11.6)	30 (16.2)	29 (15.5)	380 (11.9)	x^2 = 9.16, p = .689
25-29 n (%)	26 (26.8)	86 (30.3)	100 (30.9)	48 (25.7)	973 (30.4)	
30-34 n (%)	38 (39.2)	120 (42.2)	135 (41.7)	71 (38.0)	1,282 (40.1)	
35+ n (%)	18 (18.6)	45 (15.9)	59 (18.2)	39 (20.9)	565 (17.7)	

Pearson's chi-squared tests (x^2) used to highlight differences between participant demographics and the varying trajectories of depression symptoms for individuals with all the risk factors.

Risk factors included in this analysis were: sex, the polygenic risk score for depressive symptoms (PRS), postnatal depression, cruelty to the mother, childhood anxiety and bullied at age 10.

© 2019 Kwong ASF et al. *JAMA Network Open.*

eTable 7. Matrix of Correlations Between Risk Factors

	Sex	Polygenic Risk Score	Postnatal Depression	Cruelty to Mother	Anxiety	Bullied
Sex	1	.	.	.	.	.
Polygenic Risk Score	-0.015 (*P* = 0.36)[c]	1	.	.	.	.
Postnatal Depression	0.016 (*P* = 0.66)[b]	0.032 (*P* = 0.06)[c]	1	.	.	.
Cruelty to Mother	-0.018 (*P* = 0.60)[b]	0.039 (*P* = 0.02)[c]	0.322 (*P* < 0.001)[b]	1	.	.
Anxiety	0.017 (*P* = 0.33)[c]	0.016 (*P* = 0.33)[a]	0.125 (*P* < 0.001)[c]	0.067 (*P* < 0.001)[c]	1	.
Bullied at Age 10	-0.147 (*P* < 0.001)[b]	0.048 (*P* = 0.005)[c]	0.095 (*P* = 0.02)[b]	0.142 (*P* = 0.002)[b]	0.06 (*P* = 0.001)[c]	1

Correlations presented with *P* values for correlations in parenthesis.
Sex was coded as 0 for males and 1 for females. The PRS was standardised to have a mean of 0 and a SD of 1. Postnatal depression, cruelty to mother and bullied at age 10 were coded as 0 for no and 1 for yes. Anxiety was coded between 0-12, with greater scores corresponding to worse childhood anxiety.
[a] Analysis was conducted using Pearson's correlations.
[b] Analysis was conducted using Tetrachoric correlations.
[c] Analysis was conducted using Point-Biserial correlations and verified using Pearson's correlations.

© 2019 Kwong ASF et al. *JAMA Network Open.*

eTable 8. Adjusted Univariate Associations of All Risk Factors With Trajectories of Depressive Symptoms

Multinomial Odds Ratios (ORs) [Lower, Upper 95% CIs]					
	Childhood persistent vs. Stable low	Early-adult onset vs. Stable low	Adolescent limited vs. Stable low	Childhood-limited vs. Stable low	Omnibus *P*-value
**Sex (n=9,394)**[a]					
Female	6.13 [3.92, 9.58]	2.49 [1.97, 3.14]	4.5 [3.33, 6.07]	1.28 [0.96, 1.7]	<.001
**Genetics (n=6,309)**[a]					
Polygenic Risk Score	1.53 [1.27, 1.84]	1.2 [1.04, 1.38]	1.09 [0.94, 1.27]	1.04 [0.89, 1.23]	<.001
**Early Life (n=6,345)**[b]					
Postnatal Depression	2.14 [1.28, 3.57]	1.99 [1.35, 2.94]	1.2 [0.72, 1.99]	1.87 [1.17, 2.99]	<.001
Cruelty to Mother 2-4 Years	1.91 [1.16, 3.16]	1.73 [1.05, 2.85]	1.78 [1.2, 2.63]	1.3 [0.8, 2.12]	<.001
**Childhood (n=4,733)**[c]					
Anxiety at 7.6 Years	1.35 [1.22, 1.5]	1.14 [1.04, 1.24]	1.13 [1.03, 1.24]	1.22 [1.08, 1.37]	<.001
Bullied at 10 Years	4.94 [2.84, 8.6]	1.69 [1.15, 2.5]	1.89 [1.22, 2.91]	7.58 [5.0, 11.51]	<.001

[a] Analysis was not adjusted for any confounders or risk factors.
[b] Analysis included postnatal depression and cruelty to mother and the following confounders: biological sex, maternal age at birth, maternal socioeconomic status at birth, maternal educational attainment at birth and parity.
[c] Analysis included anxiety and bullying and was adjusted for the following confounders: biological sex, maternal age at birth, maternal socioeconomic status at birth, maternal educational attainment at birth and parity.

© 2019 Kwong ASF et al. *JAMA Network Open.*

eTable 9. Unadjusted Multivariate Associations of All Risk Factors With Trajectories of Depressive Symptoms

Multinomial Odds Ratios (ORs) [Lower, Upper 95% CIs][a]					
	Childhood persistent vs. Stable low	Early-adult onset vs. Stable low	Adolescent limited vs. Stable low	Childhood-limited vs. Stable low	Omnibus *P*-value
**Sex**					
Female	6.09 [2.91, 12.74]	2.22 [1.55, 3.19]	5.9 [3.6, 9.66]	1.88 [1.23, 2.89]*	<.001
**Genetics**					
Polygenic Risk Score	1.54 [1.16, 2.02]	1.28 [1.08, 1.52]	1.12 [0.93, 1.35]	1.05 [0.86, 1.28]	.002
**Early Life**					
Postnatal Depression	2.56 [1.3, 5.02]	1.98 [1.2, 3.28]	1.18 [0.64, 2.2]	1.69 [0.9, 3.18]	.008
Cruelty to Mother 2-4 Years	1.82 [0.82, 4.05]*	2.17 [1.36, 3.46]	2.13 [1.33, 3.42]	1.07 [0.54, 2.12]	<.001
**Childhood**					
Anxiety at 7.6 Years	1.26 [1.12, 1.4]	1.12 [1.02, 1.23]	1.1 [1, 1.21]	1.27 [1.14, 1.41]	<.001
Bullied at 10 Years	4.23 [2.27, 7.89]	1.71 [1.12, 2.6]	1.37 [0.84, 2.25]*	7.55 [4.86, 11.71]	<.001

[a] Analysis was not adjusted for any confounders.

* Indicates a substantive difference from the univariate model.

© 2019 Kwong ASF et al. *JAMA Network Open.*

eReferences

1. Euesden J, Lewis CM, O'Reilly PF. PRSice: Polygenic Risk Score software. *Bioinformatics.* 2015;31(9):1466-1468.
2. Okbay A, Baselmans BM, De Neve JE, et al. Genetic variants associated with subjective well-being, depressive symptoms, and neuroticism identified through genome-wide analyses. *Nat Genet.* 2016;48(6):624-633.
3. Taylor AE, Jones HJ, Sallis H, et al. Exploring the association of genetic factors with participation in the Avon Longitudinal Study of Parents and Children. *Int J Epidemiol.* 2018.
4. Peyrot WJ, Van der Auwera S, Milaneschi Y, et al. Does Childhood Trauma Moderate Polygenic Risk for Depression? A Meta-analysis of 5765 Subjects From the Psychiatric Genomics Consortium. *Biol Psychiatry.* 2017.
5. Wolke D, Woods S, Schulz H, Stanford K. Bullying and victimisation of primary school children in South England and South Germany: Prevalence and school factors. *British Journal of Psychology.* 2001;92:673-696.
6. Costello DM, Swendsen J, Rose JS, Dierker LC. Risk and protective factors associated with trajectories of depressed mood from adolescence to early adulthood. *J Consult Clin Psychol.* 2008;76(2):173-183.
7. Whalen DJ, Luby JL, Tilman R, Mike A, Barch D, Belden AC. Latent class profiles of depressive symptoms from early to middle childhood: predictors, outcomes, and gender effects. *J Child Psychol Psychiatry.* 2016;57(7):794-804.
8. Yaroslavsky I, Pettit JW, Lewinsohn PM, Seeley JR, Roberts RE. Heterogeneous trajectories of depressive symptoms: adolescent predictors and adult outcomes. *J Affect Disord.* 2013;148(2-3):391-399.
9. Muthen B, Muthen LK. Integrating Person-Centered and Variable-Centered Analyses: Growth Mixture Modeling With Latent Trajectory Classes. *ALCOHOLISM: CLINICAL AND EXPERIMENTAL RESEARCH.* 2000;24(6).
10. Schwarz G. Estimating the dimension of a model. *The Annals of Statistics.* 1978;6(2):461-464.
11. Lo Y, Mendell N, Rubin D. Testing the number of components in a normal mixture. *Biometrika.* 2001;88:767-778.
12. Heron J, Croudace T, Barker E, Tilling K. A comparison of approaches for assessing covariate effects in latent class analysis. *Longitudinal and Life Course Studies.* 2015;6(4).
